# Bias Due to Changes in Specified Outcomes during the Systematic Review Process

**DOI:** 10.1371/journal.pone.0009810

**Published:** 2010-03-22

**Authors:** Jamie J. Kirkham, Doug G. Altman, Paula R. Williamson

**Affiliations:** 1 Centre for Medical Statistics and Health Evaluation, University of Liverpool, Liverpool, England; 2 Centre for Statistics in Medicine, University of Oxford, Oxford, England; Memorial Sloan-Kettering Cancer Center, United States of America

## Abstract

**Background:**

Adding, omitting or changing outcomes after a systematic review protocol is published can result in bias because it increases the potential for unacknowledged or *post hoc* revisions of the planned analyses. The main objective of this study was to look for discrepancies between primary outcomes listed in protocols and in the subsequent completed reviews published on the Cochrane Library. A secondary objective was to quantify the risk of bias in a set of meta-analyses where discrepancies between outcome specifications in protocols and reviews were found.

**Methods and Findings:**

New reviews from three consecutive issues of the Cochrane Library were assessed. For each review, the primary outcome(s) listed in the review protocol and the review itself were identified and review authors were contacted to provide reasons for any discrepancies. Over a fifth (64/288, 22%) of protocol/review pairings were found to contain a discrepancy in at least one outcome measure, of which 48 (75%) were attributable to changes in the primary outcome measure. Where lead authors could recall a reason for the discrepancy in the primary outcome, there was found to be potential bias in nearly a third (8/28, 29%) of these reviews, with changes being made after knowledge of the results from individual trials. Only 4(6%) of the 64 reviews with an outcome discrepancy described the reason for the change in the review, with no acknowledgment of the change in any of the eight reviews containing potentially biased discrepancies. Outcomes that were promoted in the review were more likely to be significant than if there was no discrepancy (relative risk 1.66 95% CI (1.10, 2.49), p = 0.02).

**Conclusion:**

In a review, making changes after seeing the results for included studies can lead to biased and misleading interpretation if the importance of the outcome (primary or secondary) is changed on the basis of those results. Our assessment showed that reasons for discrepancies with the protocol are not reported in the review, demonstrating an under-recognition of the problem. Complete transparency in the reporting of changes in outcome specification is vital; systematic reviewers should ensure that any legitimate changes to outcome specification are reported with reason in the review.

## Introduction

The systematic review process has been developed to minimise biases and random errors in the evaluation of healthcare interventions, using precise and explicit methods [1]. Cochrane systematic reviews are internationally recognised as among the best sources, if not the best source, of reliable up-to-date information on health care interventions [2], [3].

One of the key components of a well-formulated review question is the specification of the particular outcomes of interest. Cochrane reviews should include all important outcomes that are likely to be meaningful to clinicians, patients and policy makers. Including all the important outcomes in a review will highlight gaps in primary research and encourage trialists to address these gaps in future studies. The Cochrane Handbook provides guidelines for specifying outcomes [4]. It states that there should generally be no more than three specified *primary* outcomes which should normally include at least one potential benefit and at least one potential area of harm. Non-primary outcomes should be listed as a limited number of *secondary* outcomes. Secondary outcomes, for example surrogate measures, should be used to help explain effects and should not be considered as main outcomes as they are less important than clinical endpoints for informing decisions.

Preparing a review is a complex process and can often require many decisions and judgements. Before a review begins, a protocol should be developed to establish in advance the methods that will be used. The protocol is an essential component when conducting a systematic review. It ensures that the review could be replicated by independent researchers and it reduces the risk of bias through explicitly stating *a priori* hypotheses and methods which should be determined without prior knowledge of results. Clearly, when no protocol is available, any such bias may go undetected.

In an individual RCT, outcome reporting bias (ORB) has been defined as the selection for publication of a subset of the original recorded outcome variables based on the results [5]. It is equally important to assess the potential for outcome reporting bias at the systematic review level. The purpose of this study is to identify inconsistencies between outcomes published in review protocols and in the associated published reviews, in relation to the potential bias such changes may introduce. Making changes after seeing the results for included studies can lead to biased and misleading results if the importance of the outcome (primary or secondary) is changed on the basis of those results. Two previous similar studies have investigated the prevalence of discrepancies between outcome definitions in published protocols and their associated reviews [6], [7] but our study adds information on the reasons for discrepancies, enabling an assessment of the potential for bias. Finally, we discuss the potential seriousness of the biases outlined in our findings along with recommendations to overcome the problems encountered.

## Methods

As part of a larger project investigating the prevalence and impact of outcome reporting bias in randomised trials on systematic reviews [8], discrepancies between specified protocol and review outcomes were also assessed in 309 new reviews published on the Cochrane Library between Issue 4, 2006 and Issue 2, 2007. Twelve reviews from the Cochrane Methodology Review Group were excluded leaving a total of 297 reviews to be assessed.

If no protocol had been published on the Cochrane Library, reviewers and Collaborative Review Groups (CRGs) were asked to provide a reason for this.

Two investigators (JJK, SD) independently examined the full protocol to determine whether the protocol specified no, one, or more than one primary outcome. Any discrepancies between the assessments were resolved through discussion. A protocol was said to have specified no primary outcome if outcomes were listed, but there was no indication which of these listed outcomes were the primary, the main or most important. The process was then repeated for the reviews. We then compared the protocol primary outcome(s) with those reported as primary in the published review, and any discrepancies (additions, omissions or changes) were noted. The review text was examined to see if (i) a declaration of the change from the protocol was made and (ii) an explanation for this change was given. When no indication of change was provided in the review, review authors were contacted and asked for the reason for the change.

Inconsistencies between protocol and review primary outcomes were classified as follows: (a) *inclusion* of at least one new primary outcome in the review that was not specified at all (i.e. as either a primary or secondary outcome) in the protocol, (b) *exclusion* of at least one primary outcome in the review that was listed as a primary outcome measure in the protocol and (c) *change* in the primary outcome(s) specified in the protocol and review. If a *change* in primary outcomes had occurred, this was classified as either an *upgrade* or *downgrade*. An *upgrade* occurred if a secondary protocol outcome was promoted to a review primary outcome and a *downgrade* occurred if a protocol primary outcome was demoted to a review secondary outcome. An *upgrade* also occurred if the review specified primary outcome(s) but the same outcomes listed in the protocol were not listed with any order of importance, i.e. primary or secondary. A *downgrade* also occurred if the protocol specified primary outcome(s) but the same outcomes listed in the review had no order of importance. It is possible that both upgrades and downgrades could occur in a single review if primary and secondary outcome measures are swopped over between protocol and review. Similarly inclusions and exclusions could occur in the same review. Discrepancies were classified and discussed with the reviewer until the final overall classification was agreed for each discrepancy. Our findings were fed back to the relevant CRG and lead reviewers.

Meta-analysis results were extracted for each primary review comparison. The primary review comparison was selected for each review according to the following hierarchy by selecting that which met the first of the following criteria: (1) an intervention comparison described in the protocol as the primary review comparison; (2) the first intervention comparison mentioned in the objectives of the protocol; (3) an intervention comparison described in the review as the primary review comparison; (4) the first intervention comparison mentioned in the objectives of the review; (5) the intervention comparison used in the first meta-analysis presented in the review.

The relative risk of obtaining a significant result for *inclusions/upgrades*, and then *downgrades*, compared to meta-analyses with no discrepancies was estimated. If a protocol outcome was *included* or *upgraded* to a review primary outcome, and the meta-analysis for that outcome gave a significant pooled effect estimate (p<0.05), then this would increase our suspicion of bias since the reason for the inclusion/upgrade could have been influenced by the significance of the result. If this hypothesis were true then we would expect the likelihood of a significant meta-analysis result to be higher for inclusions/upgrades when compared to meta-analysis results with no discrepancies. Conversely, if an outcome was downgraded in the review then our suspicion would be raised that this decision had been influenced by a non-significant (p>0.05) pooled effect estimate. If this hypothesis were true then we would expect the risk of a significant result to be lower for downgrades when compared to meta-analysis results with no discrepancies.

## Results

### Missing protocols

Eight percent (24/297) of reviews did not have a protocol sourced next to the review under the “Protocol and previous versions” section on the Cochrane Library. The reason was not provided by two lead review authors. Seven (2% of 295 reviews) did not have a protocol: five reviewers went straight from registered title to review and two reviews were published by an alternative source and were later updated and developed into a Cochrane review using Cochrane guidelines. For the remaining 15 reviews, the reviewer authors sent a copy of the protocol. These protocols were missing from the “Protocol and previous versions” section of the Cochrane Library because a) the review was split into a number of separate reviews and only one protocol was registered (9 reviews), b) a draft protocol was accepted by the Cochrane Review Group (CRG) but was not registered on the Cochrane Library as it was never formally published (4 reviews) and c) the reviewer thought the protocol was registered on the Cochrane Library but its source location could not be found (2 reviews). For this last category, the CRG was contacted and the protocols had been withdrawn from the Library on the advice of the Collaboration because they were seen to be out of date. Thus 288 protocol-review pairs were available for study.

### Comparison of outcome measures

**Figure 1:**
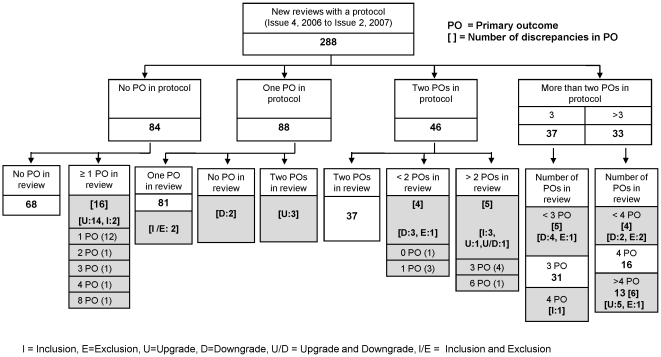
A flow diagram showing the discrepancies between protocol and review primary outcomes. The shaded areas indicate where the discrepancies were found.

Figure 1 shows the breakdown of the number of primary outcomes specified in the protocol and review for the cohort of 288 reviews where protocols existed. The median number of primary outcomes specified in the protocol and review was the same: median 1, IQR (0,2). Twenty nine percent (84/288) of protocols made no distinction between the primary and secondary outcomes and 68 of the 84 associated reviews made no distinction either. Sixty-four (22%) of 288 reviews contained a discrepancy in at least one outcome measure of which 48 (75%) were attributable to changes in the specification of at least one primary outcome measure. The 48 discrepancies in the primary outcome are identified in Figure 1, however the remaining 16 discrepancies which were not in primary outcomes are not shown in this figure.

After contacting reviewers with a discrepancy in the protocol/review primary outcome(s), 34/48 (71%) lead reviewers replied but only 28 (58%) could recall a reason for the discrepancy (Table 1). From the reasons provided, it is clear that there is potential for bias in at least 29% (8/28) of reviews with discrepancies, where changes were made after knowledge of all results.

**Table 1:** Reasons for discrepancies in primary outcome measures.

				Change			
Reasons for discrepancy between primary outcome(s) specified in the protocol and the review	Inclusion	Exclusion	Inclusion and Exclusion	Upgrade	Downgrade	Upgrade and Downgrade	Number of reviews
Recommendation by editors/peer reviewers	-	1	1	3	2	-	**7**
Recognition of the importance of the outcome **before** reading the results for the included trials	-	-	-	5	2	-	**7**
†Recognition of the importance of the outcome **after** reading the results for the included trials	3	-	-	2	2	-	**7**
†Outcome reflects the same domain as another outcome specified. Decision made **after** reading the results for the included trials	-	1	-	-	-	-	**1**
No results reported in the literature	-	1	-	-	1	-	**2**
Change in author from protocol/review – change reflects opinion of the importance of the outcome from another expert	1	1	-	2	-	-	* **4**
Reviewer responded but could not recall reason for discrepancy	-	-	-	6	-	-	**6**
No response from authors	2	1	1	5	4	1	**14**
**Total**	**6**	**5**	**2**	**23**	**11**	**1**	**48**

† Reasons where potential bias was suspected.

* Delay between publication of the review and protocol for these four reviews: 27 months, 66 months, 75 months and 99 months (median for all 288 reviews was 24 months).

Sixty seven percent of the review discrepancies involved either an *inclusion* or an *upgrade* where at least one primary outcome was added to the review that was not mentioned in the protocol. From a total of 245 meta-analyses of primary review comparisons from 148 reviews, 85 of these analyses showed a significant result (p<0.05) in favour of the intervention while 160 analyses showed a non-significant result (p>0.05). Table 2 provides a comparison of the significance of the results depending on whether an *inclusion*, *upgrade* or *downgrade* was found. There was an increased risk of obtaining a significant result in the meta-analysis if the discrepancy was either an *inclusion* or an *upgrade* than if there was no discrepancy (relative risk 1.66 95% CI (1.10, 2.49), p = 0.02). When considering protocol primary outcomes that were changed to secondary outcomes or not listed with any order of importance in the review (*downgrades*), there was no discernable decreased risk of obtaining a significant result in the meta-analysis than if there was no discrepancy (relatively risk 0.95 95% CI (0.41, 2.19, p = 0.90).

**Table 2:** A comparison of the significance of meta-analysis results for primary review comparisons between outcomes that are either inclusions, upgrades or downgrades in the review and those outcomes where there are no discrepancies between protocol and review.

		Significance of meta-analysis result		
		Significant result (p<0.05)	Non-significant result (p>0.05)	
Type of discrepancy	Inclusions	3 (3)	2 (2)	5 (5)
	Upgrades	11 (7)	10 (10)	21 (17)
	Downgrades	4 (3)	9 (6)	13 (9)
	No discrepancy	67 (46)	139 (71)	206 (117)
		85 (59)	160 (89)	245 (148)

Numbers in parentheses represent the number of reviews affected by each discrepancy.

A thorough examination of the review text revealed that only 4(6%) of the 64 reviews with deviations from the protocol outcome specifications described the reason for the changes in the review. In all four of these reviews, the reason for the discrepancy reported in the review matched the reason provided when the reviewer was contacted. None of these acknowledgments were from the eight reviews containing potentially biased discrepancies.

## Discussion

Our study has shown substantial agreement between Cochrane reviews and prior protocols over the last few years but also highlights a concern about a previously unreported source of bias.

Discrepancies between the specification of outcome measures in protocols and reviews have been described previously but, to our knowledge, this is the first study that has sought the reasons for such discrepancies. Bias was suspected in 29% (8/28) of reviews with discrepancies in specified primary outcomes, where changes were made after knowledge of all results. None of these reviews reported the reason for the discrepancy, demonstrating an under-recognition of the problem.

This study provides evidence that outcome reporting bias, as a result of changing the defined importance of an outcome, occurs in the systematic review process as well as for individual randomised controlled trials [9]. A systematic review of the empirical evidence of outcome reporting bias in trial primary outcomes found that 40–62% of studies have at least one primary outcome that was changed, introduced, or omitted and that outcomes that are statistically significant have higher odds of being fully reported (range of odds ratios: 2.2 to 4.7) [10]. Our study shows that ORB does not appear to be limited to individual trials but also occurs in systematic reviews.

### Comparison with other studies

Higgins *et al.* [11] reported 28% (11/39) reviews with unpublished protocols in Issue 2 1999, while Silagy *et al.* [6] reported 29% (16/66) protocols missing from the Cochrane Library from Issue 3, 2000. A later study reported 12% (14/120) missing protocols from reviews published in 2005/06 (Parmelli *et al.* [7]). However, none of these studies mentioned whether review authors were contacted to enquire if a protocol was available on request. Our study found 8% (24/297) of reviews did not have a protocol on the Cochrane Library, but after obtaining unpublished protocols from review authors, we found that only 2% of reviews in the study cohort had no protocol. Systematic reviews that are not Cochrane reviews are less likely to have a protocol and so any *post hoc* changes cannot be identified if not indicated in the review. Only 11% of non-Cochrane therapeutic reviews in 2004 mentioned a protocol [3].

In this current study we found that 25% (75/297) of reviews did not distinguish between primary and secondary outcomes, an improvement over a study that reported a rate of 47% for reviews between 1998 and 2005 [12].

Discrepancies between any outcomes specified in the protocol and the review was found in nearly a quarter (22%) of the reviews we examined. The majority of these (75%) were attributable to changes in the specification of primary outcome measures. These results show improvement over an 81% discrepancy rate reported for Cochrane reviews published in 2000 [6] and 47% for reviews published in 2005/06. [7].

Non-significant meta-analysis results were found when outcomes were *downgraded* while *upgrades* or *inclusions* favoured statistically significant outcomes when compared to the results where no discrepancy in outcome definition was found. In addition, two reviews *downgraded* or *excluded* the protocol primary outcome measures from the review because no results were reported in the literature.

### Conclusions and policy implications for systematic reviews

Our study shows substantial improvements in Cochrane reviews over time with respect to outcomes. There is still room to increase the quality however, and we would recommend the following. The reviewer, and especially the CRG, should ensure that the policy of writing and registering a carefully designed protocol prior to the start of each review is followed. The Cochrane Handbook recommends that up to a maximum of seven desirable and undesirable outcomes (listed in order of importance) that are essential for decision-making should be decided by reviewers during protocol development and included in the ‘Summary of findings’ section of the review. CRGs should be encouraged not to allow reviewers to proceed with the review before a protocol has been reviewed by an appropriate external review panel. When one protocol is written for multiple reviews, it should be made clear where the protocol is located on the library. Most importantly, systematic review protocols should be made publicly available to deter, and enable the identification of, outcome reporting bias and unacknowledged *post hoc* amendments to pre-specified outcomes.

The Cochrane Handbook acknowledges that review authors should be alert to the possibility that the importance of an outcome may only become known after the protocol was written or the analysis was carried out, and should take appropriate actions to include these in the review. There is still a need for reviewers to describe the legitimacy of adding or changing outcomes after the protocol was published in order to prevent any suspicion of bias as well as adhering to these current Cochrane guidelines. Moreover, outcome definitions should not be changed because they are more frequently addressed in the studies that are being reviewed, nor changed on the basis of observed magnitude of effect.

As well as being aware of potential outcome reporting bias in the systematic review process, it is important for reviewers to assess the impact of this type of bias in the clinical trials within the review. A systematic empirical study of the impact of outcome reporting bias in randomised controlled trials on the results of systematic reviews revealed that a third of Cochrane reviews (96/283, 34%) contained at least one trial with high ORB suspicion for the review primary outcome [8]. Moreover, ORB was suspected in a single primary review outcome in 14% (359/2486) of assessable randomised controlled trials [8]. The adoption of the new Cochrane risk of bias tool, which includes a judgment of the risk of selective outcome reporting for included studies, should also help to raise awareness of outcome reporting bias.

By looking at only Cochrane systematic reviews, we suspect that our study underestimates bias due to changes in outcome specification during the systematic process. Cochrane reviews are not only monitored by a CRG but also the Cochrane Handbook provides guidelines which offer some protection against this type of bias [4]. A recent commentary calls for the registration of all systematic reviews [13]. Such a registry may reduce publication bias in reviews as well as enhancing transparency and avoiding duplication of effort. In addition, the recently published PRISMA (Preferred Reporting Items for Systematic Reviews and Meta-Analyses) statement has evolved to help ensure the clarity and transparency of reporting of systematic reviews [14]. The statement specifically asks review authors to report on registration and availability of their systematic review protocol in order to reduce the risk of flawed reporting of systematic reviews that may lead to bias.

The review authors are working with reviewers/CRGs to improve the reporting of outcome data and to reduce bias in systematic reviews as an ongoing investigation. Any problems or suspicions of potential sources of outcome reporting bias are being fed back to reviewers CRG and thus far the feedback has been well received.
